# Surgery

**Published:** 1836-10

**Authors:** 


### SURGERY.

*On the Diseases of the Lymphatic System.* By A. Velpeau, Professor of the Faculty of Medicine; Surgeon at La Charité.

### I. *On Inflammation of the Lymphatic Vessels.*

When in contact with diseased products, lymphatic vessels may imbibe matters capable of exciting inflammation in themselves. In one class of cases, these vessels are in contact with morbid parts or deposits which are protected from, and in another with those which are exposed to, the influence of the external air. In an ulcer, for instance, all the products of inflammation undergo various and rapid modifications, and these are in some cases rendered actually poisonous. The absence of such a condition, as in disease protected from the air, must diminish the activity of the exciting cause, and render inflammation from the absorbed matter much less probable. Inflammation of the lymphatics *(angioleucitis)* is, therefore, much more rare where there is not external solution of continuity than when this exists.

Among the causes, independent of the influence of the external air, which may give rise to angioleucitis, may be mentioned all forms of inflammation of parenchyma, glands, cellular tissue; pus and other fluids, however deposited; tubercular, cancerous, and the majority of morbid productions or degenerations. Whenever there is exposure to the atmosphere, its influence is superadded; as in the majority of cutaneous diseases, fistulous openings, and in solutions of continuity of mucous membranes. These causes must be considered as only predisposing in the majority of instances, or as requiring some other condition before they become excitants. The old and the young are more subject to their influence than the adult and those at the middle period of life; the constitution in which cellular tissue abounds, than the robust and the fibrous; the debilitated from any cause, than the healthy and strong.

The symptoms are local or general; the local symptoms are superficial or deep.

*Local symptoms.* When the subcutaneous vessels are affected, the cause may almost always be found in a solution of continuity, inflammation or suppuration of the integuments. Lines of various breadth, following the course of the lymphatics, tortuous, irregular, and interlaced, or patches of red, rosy, or violet colour, soon appear in the region of the irritant cause, or at some distance above. Inflammatory patches next follow, and eventually spread over the unchanged integument, until a true erysipelas is formed. Similar changes occur at a greater distance from their cause, giving the appearance of an erysipelatous inflammation united by red lines and stripes. A sharp and burning pain, much increased by touch, attends the redness. Swelling is at first but slight, and sometimes not observable even in the vessels themselves, which remain supple; but, in general, subcutaneous swelling very soon follows the appearance of redness, commonly extending irregularly both in depth and surface, and being ordinarily nodulated, but sometimes more extended. The skin, cellular tissue beneath, and neighbouring parts, appear to share in the swelling; but the inflammation attaches itself more to the vessels, the plexuses, and the lymphatic glands, than to the cellular tissue generally. The parts soon become more tense, but still an œdematous or spongy character indicates a state of infiltration, and establishes a marked distinction between the regular elasticity of phlegmon or the less resilience of erysipelas. Heat, at first considerable, does not increase in proportion to the other symptoms. Enlargement of the glands in the course of the lymphatic vessels is not always present.

When the deep series of lymphatics is first affected, pain is the earliest symptom, deep, lancinating, and fixed, extending with or soon following the inflammation, and most severe in those parts where the inflammation is most developed. A nodulated and deeply-seated swelling appears about the same period and in the same situation as the pain. Redness follows: it is less superficial and more diffuse than in the former variety. The skin is tense and shining; rather of a pale rose colour than red in the intervals of the inflamed nodules. It is the deep lymphatic glands which swell and become painful. Infiltration is more rapid and extensive, and may give rise to the idea of an inflammatory œdema; but, in general, both series of lymphatics are simultaneously or reciprocally affected, when the symptoms are mixed.

*General symptoms.* These are little modified by the series of lymphatics which happens to be affected. Slight horripilations or distinct rigors, followed by heat, dryness, and redness of skin, precede the inflammation. The pulse, always frequent, is sometimes full and strong; at others small and frequent, as in infectious fevers. Thirst, præcordial anxiety, nausea or vomiting, sleeplessness, rarely with delirium, constitute other symptoms, which, together or in part, may for two or three days precede the local symptoms. The secretions are not always disturbed. Fever increases as the angioleucitis is developed. The tongue, with a grey or yellowish coating, is smooth and red at its tip and edges: it rarely becomes dry, until an advanced period; at times it is universally rough, without always losing its grey or reddish colour. It afterwards becomes incrusted and chapped; the gums and mouth are covered with sordes; and reaction, more like ataxic than adynamic fever, terminates life.

*Terminations and Prognosis.* When the inflammation is of limited extent, and does not pass to the deep series of vessels, and when the affection that produced it improves, either spontaneously or on the use of remedies, resolution may be calculated on; otherwise, suppuration is almost inevitable. When there are numerous red patches which coalesce, and beneath, nodules of a certain thickness, suppuration, although slow, may be expected. Induration, without suppuration, is of rare occurrence, and belongs almost exclusively to the chronic form of the disease. Death may occur during the inflammatory or suppurative stage; it is to be feared, when violent general symptoms, coinciding with extensive inflammation, continue more than eight or ten days; when the extent and depth of the inflammation in an old or unhealthy subject promise abundant suppuration; when there are numerous and repeated purulent deposits; when these are formed in the viscera or serous cavities, and when the blood is altered by the matters absorbed. In some cases the symptoms are regularly progressive; in others they appear to belong to a succession of inflammatory attacks. Resolution may happen between the fourth and the tenth day; death, generally, between the eighth and the twentieth. After the thirtieth or fortieth days, induration may take place, but internal deposits, diarrhœa, and an infected state of the blood are most to be feared.

*Treatment.* Any sore that may exist should be covered with a poultice. If much arterial action be present, a large bleeding, followed by a warm bath, of an hour, should be employed. If there be redness and swelling around the sore, twenty or thirty leeches should be applied, and afterwards a roller, constantly moistened with cold water. If these means are useless, let two drachms of mercurial ointment be rubbed over and beyond the painful parts, three times during the twenty-four hours; the skin being afterwards cleaned with oil, and the bath repeated. The earliest formation of pus must be liberated by a free incision; the part to be treated by poultices or compression. If resolution do not happen, and suppuration is slow to develope itself, large blisters should be applied to all the parts inflamed: they promote either maturation or resolution, better than any other means. Purgatives should be employed, if diarrhœa or symptoms of affection of serous membranes be absent. Suppuration having ceased, but an indurated or infiltrated condition remaining, ioduretted ointment should be used, or the mercurial ointment, if the infiltration be extensive, aided by compression and baths. Diet must be strictly attended to.

*Differential diagnosis.* Angioleucitis has been confounded with phlebitis, neuritis or neuralgia, erysipelas, phlegmon, &c. [Of these, it will only be necessary to notice the author's remarks on the distinctive characters of phlebitis; a very careless observer alone would confound with inflammation of lymphatics, the other affections.]

Phlebitis is commonly announced by local symptoms, instead of by general reaction. It appears with larger, fewer, deeper and less interlaced stripes, which correspond to hard, rounded, moveable, and painful chords. When there are external patches of red, they are situated over more extensive nodules, which are less deep and hard. The patches rarely coalesce, so as to form true erysipelas. Suppuration is more rapid, and the abscesses are diffuse. These are few, and generally in the course of the large veins. Swelling is less in extent and degree; the skin is neither tense nor shining, but rather thickened and simply inflamed. *The lymphatic glands are neither swelled nor painful.* Pain is less acute, and general symptoms more adynamic than inflammatory; rarely existing until pus has formed and entered the circulation. Their development is very rapid; rigors are long, violent, and irregular; the pulse very small and unequal; the tongue becomes slimy, generally red, then blackening, before it grows dry and incrusted. There is less anxiety, agitation, heat, and thirst. Delirium and sweatings, followed by an expression of stupor, occur sooner. There is generally either diarrhœa or constipation. Inflammation may extend from the veins to the absorbents and inversely, when the symptoms are mixed.

It is well to remember that phlegmonous erysipelas may be associated with angioleucitis.

*Morbid Anatomy.* Such of the lymphatics as can be examined are thickened; internally of a milky whiteness and somewhat tomentose, but still permeable; the surrounding cellular tissue is easily broken, and infiltrated with turbid demi-concrete lymph. Where the vessels are interlaced, and opposite the valves, the disease is more developed. Pus is here generally found in the cellular tissue; the vessels are often obliterated, and lardaceous nodules constitute the commencement of abscesses. The skin, often covered with phlyctenæ, is scarred or mortified in patches, which resemble the sloughs of anthrax, and appear rather in a state of suppuration than of gangrene. Beneath the integument, the cellular tissue is in some places healthy; in others, hardened or lardaceous, at times, infiltrated with pus and turbid serum, and ulcerated at the situation of purulent deposits. The fasciæ, nerves, and muscles are but little changed. The interstitial cellular tissue is the chief seat of disease; it is everywhere infiltrated, hardened, or thickened, as beneath the skin; pus is generally situated in circumscribed abscesses. Mortification is very rare beneath fasciæ. The veins and arteries appear hardened and enlarged, owing to the infiltration of their cellular coats, from the numerous lymphatics existing in their vicinity. If the disease has lasted long, the blood is always very fluid, serous, of a reddish colour rather than black. The few coagula in the veins are diffluent, and often contain yellowish grains; and those which are formed in the arterial system are more friable than after death from purely inflammatory diseases, and are besides less homogeneous, containing lumps frequently of a yellowish, black, blue, or red colour. Pus has never been found in the heart or large vessels. The parenchymatous organs are rarely the seats of abscess by metastasis. Those which are found in the liver and lungs are more remarkable for their number than their size. Concrete masses are often found when angioleucitis has arisen from a cancerous affection. In many subjects, sero-purulent effusions into the pleuræ, peritoneum, and articulations, and partial inflammation of the viscera are observed. The brain and alimentary canal, mucous follicles and glands of Peyer, are unaffected. If there be ulceration, it is generally towards the end of the large intestines, and particularly in the rectum, unless the disease have commenced there. The appreciable internal morbid changes are far from being always in relation with the symptoms observed during life. The same remark is, in a less degree, applicable to the local changes; where the traces of inflammatory action may be trifling, although the symptoms of angioleucitis have been intense.

### II. *On Inflammation of the Lymphatic Glands.*

The Causes of Inflammation of the Lymphatic Glands, *(Adénite lymphatique,)* are (1,) *direct causes,* as external violence of every kind; and (2,) *indirect causes.* The inflammation may be transmitted in three ways: 1. By contiguity of these tissues; as when inflammation attacks primarily the neighbouring cellular tissue, and passes to the glands. 2. By the lymphatic vessels becoming primarily inflamed, and the glands secondarily. 3. By the transmission of irritating matter from a distant source. This is by far the most common cause.

It is unnecessary here to discuss the question whether the lymphatics absorb, for the fact is sufficient for the present purpose, that a morbid principle in a distant part may produce inflammation of a lymphatic gland without affecting in any appreciable way the intermediate tissues; as buboes, enlarged axillary glands, &c. demonstrate daily. All inflammations, therefore, as they all in some way modify the fluids, may produce this disease. The altered fluids are gradually taken into the circulation, and especially affect the lymphatic glands, either from the elaboration which these fluids undergo increasing their irritating qualities, or from their contact being prolonged by the delay in their progress. Thus a scratch, the smallest pustule, a burn, a chap, an excoriation, an ulcer, any inflammatory disease of the skin or cellular tissue, or of the tissues surrounding the arteries, veins, nerves, or muscles; luxations, fractures, blisters, moxas, setons, cupping; small-pox and chicken-pox, measles, scarlatina; indeed all operations which in any way divide, bruise, or injure the tissues, are causes of inflammation of the lymphatic glands. This inflammation may remain after the cause is removed, and may be set up without any trace of inflammation of the connecting lymphatic vessels. On these accounts the connexion between the original inflammation and the glandular tumours in many subjects is not discovered: other difficulties may arise from the trifling nature of the primary cause, or from its being deeply seated: it may be latent and obscure, so that the inflamed gland is the first appreciable symptom: it thus is sometimes a prelude to erysipelas, and thus it appears after great fatigue, chills, &c. Most frequently, indeed, the practitioner never thinks of connecting the enlargement of the gland with any such previous cause, and M. Velpeau takes much credit to himself for having insisted, many years ago, on the importance of this connexion so universally overlooked.

The diseases which most frequently produce this secondary inflammation of the lymphatic glands are those of the skin, mucous membranes, subcutaneous, subserous, and intermuscular cellular membrane, and of that surrounding the vessels: more indirectly diseases of the joints, bones, viscera, muscles, &c. may cause it, as well as certain foods, drinks, regimen, occupation, habits, &c. which modify in any injurious way the white fluids. Are not scrofulous tumours developed in the same way? In children, young people, and women, particularly the fair and delicate, the fluids and white tissues so predominate, or are so organized that the least cause produces changes which become speedily the source of indurations, infiltrations, inflammation of the lymphatic glands, and of other parts of the same system. During twenty years, M. Velpeau has examined 900 scrofulous persons in order to elucidate the question proposed above: in 730 either inflammation or suppuration of the skin or cellular tissue had preceded the lymphatic tumour; the disease in ninety-five others was of so long standing, that no positive information could be obtained; the remaining ninety-five were children so poor, and so neglected by their parents, that the existence of one of these causes, although not admitted, might reasonably be supposed.

If it was possible to establish the fact that the strumous swelling of the lymphatic glands is almost constantly secondary, that it is at first the symptom only of a pathological action situated elsewhere, or the proof that the white fluids are unusually irritating, considerable progress would be made in the etiology of scrofula. It would then no longer be necessary to suppose a particular vice or fault of the constitution, an hereditary principle in the economy, as scrofulous diseases would arise from certain states of the organization easily comprehended.

Infancy is most exposed to these diseases, as the skin and mucous membranes are most frequently diseased. Among children, those whose tissues are pale and gorged with fluids are most liable to be affected, because their integuments are more susceptible of impressions, and their fluids more easily modified. The effects of poverty, bad food, want of clothing, moist cold, &c. in exciting scrofulous enlargements of the glands, are in the same way explained. Dentition, ulceration of the ears, affections of the scalp, coryza, explain the frequency of strumous tumours about the neck and jaw. Catarrhs, hooping cough, measles, scarlatina, frequent attacks of bronchitis, to all of which the young are subject, account for those in the chest; and affections of the intestines explain diseases of the mesenteric glands. The common infiltration of the nose and upper lip may be either the cause or the effect:—the cause, as such lesions produce enlargement of the neighbouring glands; the effect, as when these glands are diseased they at once delay the progress of the lymph from those parts from which they derive their vessels. On this hypothesis predisposing causes are not rejected, but the determining or occasional causes are explained.

Inflammation of the lymphatic glands may be acute or chronic.

1. *Acute.* The gland swells, becomes hard and painful, being often preceded by chills and febrile symptoms, the inflammation then extends to the surrounding cellular membrane. After six days to a fortnight, suppuration takes place in several circumscribed points; and, when opened, the quantity of pus is rarely in relation to the size of the tumour, being either much less or the contrary. The first is the case when the collection of pus is between the gland and the skin, the second when suppuration is established between the glands, or between them and the deeper-seated parts. The swelling never disappears suddenly after the pus is evacuated, but very slowly: the surrounding cellular tissue being first restored, and the gland secondarily.

*Terminations.* 1. *Resolution* is much more common than is admitted. It may be expected if the primary disease is abated or removed before the inflammation passes to the surrounding cellular tissues; or, in the greater number of cases, if the inflammation is confined to the gland itself. It rarely takes place if the cellular tissue becomes inflamed; and if, besides this, the skin is red, and the subjacent layers much gorged, resolution should not be expected. 2. *Suppuration* takes place in two ways. When the gland itself suppurates, it becomes infiltrated with pus, and not a true abscess: ordinarily, the surrounding cellular tissue suppurates, and the abscess differs from phlegmon only in the irregularity produced by the presence of the enlarged glands; sometimes both suppurate. Suppuration may be expected when the enlarged gland depends on a suppurating wound, or when the inflammation extends beyond the gland. 3. In the chronic stage the swelling diminishes, but a little pain and heat, with some increase of size, remain. If these glands are examined in the dead body they are found of a dirty reddish yellow, homogeneous, and hard; sometimes containing a little concrete or half-liquid pus.

*Treatment.* In the first instance, a large bleeding, if indicated by the pulse; twenty to forty leeches, or cupping two or three times in two days; poultices or emollient compresses; baths; mercurial frictions, and blisters after depletion. Syphilitic buboes, if suppuration has not taken place, are resolved most rapidly by blisters only. When suppuration has taken place, these means are useless, and poultices should be applied: at this stage it is an important question to solve whether the abscess should be opened, and what are the best means of opening it. M. Velpeau considers that the abscess should certainly be opened; for, if it is not, the pus burrows, and a large portion of skin becomes disorganized. As a general rule, the knife should be employed at an early period; if the skin is thin, livid, and denuded, either potass or the actual cautery have this advantage, that they destroy the skin at once, and leave a wound which speedily heals; but, in all other cases, the bistoury is preferable; and, even in these, it can be used to remove the loose skin. The opening should be made as soon as fluctuation is felt; for, by waiting until the abscesses are matured, the same inconveniences are experienced as result from leaving the opening to nature. Long and numerous incisions are more advisable than small and few punctures: if many points of the tumour appear softened, as if from distinct collections, M. Velpeau makes an incision through the whole length of each; when there is but one collection, one incision is to be made reaching beyond the inferior limits of the abscess, bearing in mind, that in all cases an opening should be made so large that the pus cannot stagnate.

[It must be recollected that M. Velpeau is a hospital surgeon, and that these directions for opening suppurated glands are not entirely calculated for private practice, where the "*jucunde*" is of as much importance as the "*cito.*" Long and numerous incisions in the necks of children or young ladies would be as injurious to the credit of the surgeon as to the appearance of his patients; and it is a fortunate circumstance that small punctures, if they are made when suppuration is first discovered, and care is taken to press out as much matter as possible, will generally succeed. Sir Astley Cooper, whose authority on these subjects cannot be disputed, opens such abscesses on the neck with a cataract knife, making the wound transversely so as to be hidden by the creases of the neck.]

*Archives Génerales de Medicine. Juin et Juillet*, 1835, *et Janvier*, 1836.

---

### *On Vesico-Vaginal Fistulæ, and on a new Method of Treating them.* By M. JOBERT.

THESE fistulæ are both congenital and accidental; the former, very much more infrequent than the latter, and arising from a want of development of the parts intervening between the bladder and vagina. The accidental vesico-vaginal fistulæ vary in their seat, their form, and the causes which give rise to them. These causes may act either from within the bladder upon the vagina, or inversely, or may originate within the uterus; and, lastly, these fistulæ are sometimes owing to the use of certain instruments. In the first class of causes may be mentioned, pins which penetrate from the bladder into the vagina, and calculi which destroy the partition between them. To the second belong pessaries, cancer, syphilitic ulcers. To the third, (and this is by far the most frequent,) the descent of the head of the child, and the consequent pressure upon the vesico-vaginal partition. Among the causes of the fourth class may be arranged the use of dilaters; the perforator; bruised pieces of bone; instruments applied to the vesico-vaginal septum, in order to remove calculi, &c. Of the efficacy of all these causes in producing fistulæ between the bladder and vagina there are proofs recorded by various authors.

*Pathological Anatomy.* Whether the fistulæ occupy the neck of the bladder, or are in the neighbourhood of the cervix uteri, or, as is most commonly the case, they are situated between these two points, they may be limited to the partition; but they often extend right and left, and then the vagina is consequently destroyed in a greater extent than the bladder. It may be stated, as a general rule, but not without exception, that the bladder is in these cases less open than the vagina. In the first stage there is an eschar. After an uncertain interval, by the separation of this eschar, an aperture is formed, through which the urine passes. Lined by an accidental membrane, which is continuous with that of the bladder and vagina, these fistulæ undergo changes in proportion to the length of their existence. They become dense and resistant, and establish a true continuity between the bladder and vagina. The filamentous cellular tissue which exists between the vagina and bladder becomes intimately united with them in consequence of inflammation. The blood-vessels are obliterated to the extent of a line around the cicatrix, and in the same point; the cellular, muscular, and mucous tissues of the bladder, are confounded and become dense and closely connected. The vagina, at the cicatrized part, is hard. The mucous membrane is thickened and rough: indurations take place in its parietes. When the fistula approaches the cervix uteri, the inflammation extends to the peritoneum; it gives rise to the formation of false membranes and consequent adhesions, which prevent the infiltration of the urine. The bladder, no longer a reservoir of urine, which escapes as soon as it enters this organ, contracts; and the urethra, its function being stopped, is diminished, and eventually its caliber obliterated.

*Symptoms.* The ordinary characters of urinary fistulæ are found to exist. They are round, transverse or longitudinal. The first are sufficiently common; the second are more frequent, the last are the rarest of all. The extent of these fistulæ is very variable: in some instances being only a few lines in length; in others occupying the whole partition. When the connexion between the vagina and bladder has been made by a cutting instrument, the urine immediately escapes by the former; not so, however, when the fistula is the result of a tedious labour. The partition loses its vitality, the parts swell, inflame, and there is consequent retention of urine, requiring the use of the catheter. The eschar must separate before the urine escapes. The time occupied by the separation of the eschar is variable; after two days in some, in others not until eight or ten days have elapsed. When the fistula is large, the urine escapes as soon as it is received into the bladder; but, on the contrary, when small, or when occupying the neck of the bladder, the urine escapes partially, and the patient is aware of its passage through the vagina. The position of the patient influences the flow of the urine. In some cases it ceases in the upright, in others in the horizontal posture. When the fistula occupies the neck of the bladder, the urine may be retained a considerable period, but never beyond two hours, and in this case the horizontal position is the least favorable to its escape. The relation also which is borne to the borders of the fistula by contiguous viscera modifies the passage of the urine; thus, the pressure of the uterus and of the small intestine tends to obliterate the aperture by the approximation of its sides. Walking, voiding the fæces, cough, menstruation, are all conditions, which facilitate and increase the course of the urine by the vagina. The contact of the urine with the interior of the vagina and thighs is a cause of great inconvenience, and sometimes of worse effects. It is doubtless an occasional cause of cancer of the generative organs; it produces ulcers of the vagina and thighs, and a constant state of turgidity of these parts, with erysipelas and eruptions of various characters. The common effect of it is to render the individual so disgusted with herself that she lives entirely alone. Calcareous concretions may accompany vesico-vaginal fistulæ, deposited by the urine, and they produce great pain and ulcerations of various extent. Some patients are the subjects of obstinate constipation; possibly owing to a contracted state of the fibres of the rectum, in consequence of the incipient escape of the urine by the vagina. These fistulæ may be accompanied by indurations, by circular contraction of the vagina, by bands across the vagina, by destruction of the anterior and posterior parietes of the vagina. It is said, that the urine may be poured into a species of sack, formed between the laminæ of the vesico-vaginal partition; but this, of which there is only one case on record, must be necessarily very rare.

*Diagnosis.* When the fistula is formed, this is sufficiently easy; but it is less easy to say with certainty whether or no it will occur. It is difficult to ascertain whether gangrene has happened during laborious delivery; if ischuria should follow, it cannot be until after the separation of the eschar that it can be known to depend on the formation of fistula. Now, the time for the separation of the eschar is very variable; sometimes not until the tenth or twelfth day after delivery, and a case is mentioned in which a month elapsed before the urine made its escape by the vagina. Occasionally, after labour the neck of the bladder does not quickly recover its contractile energy. Some women, indeed, throughout life, can with difficulty retain their urine, which escapes by drops when they lie down. This urine, passing into the vagina, and again escaping, presents exactly the same appearance as a fistula. The introduction of a catheter will immediately decide the point, if it be not settled by the return of the parts to their normal functions. To distinguish a communication between the vagina and bladder, pass the finger along the anterior paries of the former, and any loss of substance will thus be recognised; a coloured injection may then be forced through a catheter into the bladder, and, the finger being within the vagina, will feel the escape of the fluid by the aperture. A sound introduced into the bladder may be felt through the fistulous opening, by passing the finger into the vagina, or a sound may be passed into the bladder through the fistula in the vagina. The speculum is a valuable instrument by which to ascertain the form, situation, and extent of the fistulous structure; and, by its means, fistulæ may be detected which might escape other means of investigation.

*Treatment.* The transverse fistulæ are in general considered as the most difficult to cure. The indications of treatment are different, according to the object which is aimed at; whether it be the complications of the disease, or whether palliative means only are employed, or an attempt is made at a radical cure.

*Treatment of Complications.* It is necessary to moderate the inflammation of the bladder and vagina by appropriate means; by baths, enemata, emollient cataplasms, copious demulcent drinks. The gravelly incrustations which sometimes take place in the vagina must be removed by frequent injections or other means, and proper medicines be employed, as much as possible, to prevent their formation, as they would much interfere with the success of an operation. Every effort must be made to produce a favorable condition of the general health, previous to any operation being performed.

*Curative Treatment.* To effect a radical cure, two principal methods have been followed; one by mediate or secondary reunion; the other by immediate reunion. Several procedures have been recommended, in order to effect the immediate or secondary reunion: 1, cauterization; 2, the method of Desault; 3, that of Jules Cloquet. The means employed to produce immediate union are—1, suture; 2, the approximation of the borders of the fistula by uniting instruments; 3, the method of the present author, and which is an application of élytroplasty. We pass over the description of the various means which have been employed with such various degrees of success, to the method of M. Jobert, to which he was led by a careful examination of the previous methods of treatment by surgeons, a conviction of their manifold imperfections, and the serious inconveniences which are connected with them. The object of M. Jobert is to cut a flap, to supply the lost substance; to keep it in its place, and to reestablish the proper current of urine. It is necessary, 1, to renew the borders of the fistula; 2, to cut a flap; 3, to employ a suture; 4, to reestablish the course of the urine. By careful traction, the vesico-vaginal septum may be drawn out, and the borders of the fistula be there incised. This may sometimes be effected by a finger introduced into the fistula. M. Jobert commonly employs the hooks of Museux, by which he seizes either border of the fistula, and pares it, beginning with the posterior, and finishing with that which is nearer to the urethra. For this, a probe-pointed bistoury, or the scissors employed by M. Roux in palate-suture, may be used. The whole border of the fistula must be carefully pared. One of the valves of the author's speculum is advantageous to guide the hooks to the lips of the fistula. The flap may be taken from the external labia, from the adjoining parts, or from both. Its size must be proportioned to that of the fistula. It is better that it should be somewhat larger, (but not much,) as it then possesses more vitality, and is less likely to mortify. The hairs must be first shaved, and the flap, when cut, so twisted on itself that the bleeding surfaces may be brought in contact. The pedicle should be of considerable size, and attached to the point which is nearest to the vagina. It should be of sufficient length, not at all to pull the vagina, and to allow freedom to its circulation. It must not consist solely of skin; in this case, gangrene easily occurs; but some of the subjacent tissues must be removed at the same time. The operation must be delayed if the health is not good, or until any ulcerations of the vagina have been cured. Should there be any bands crossing the vagina, they must be divided.

In order to cut the flap, the integuments of the labium being stretched with one hand, an incision must be made on its external side, from above downwards; and, when this has been carried to a sufficient extent, the knife being turned, must be carried to the internal surface of the labium, on a level with the commencement of the incision. The flap has then a rounded extremity, and is fitted for large fistulæ. The flap may be made by two incisions instead of one; each one commencing above, and uniting beneath, at a more or less acute angle. This is more appropriate to smaller fistulæ. Then dissect the skin, and with it some of the subjacent tissues, from without inwards, and from the extremity towards the pedicle of the flap. Any arteries which bleed on the surface of the wound should be tied. In women whose vitality is very active the flap contracts into numerous folds. The pedicle should be somewhat larger than the flap itself; for it is essential for its union with the borders of the fistula that its circulation should be free. As the flap is disposed to contract during its suppuration, M. Jobert prefers to take it not only from the labium, but from the neighbouring parts, by extending his incisions. Where the woman is fat, it is better to make the flap entirely from the larger labium; for the object should be to obtain a flap consisting of skin and the subjacent vascular tissue, rather than of much adipose tissue. In order to introduce the flap into the fistula, M. Jobert doubles it up, and traverses its extremity by a waxed thread. He then introduces a catheter into the bladder, through the urethra, and directs its point into the vagina through the fistula. He then passes the two ends of the thread through the eyes of the catheter. Withdrawing this, he next disengages the thread from the instrument; and then, by pressing with one hand upon the flap, and by drawing the thread with the other, he brings it to the fistula.

The next step in the operation is the suture. The finger being introduced along the flap, a curved needle mounted on the needle-bearer employed in palate-suture, is then passed above it, or the needle may be directed by the hand alone. By one effort the needle is passed through the flap and the borders of the fistula, and is withdrawn with its thread by means of a pair of dressing forceps. The same suture is then made at the opposite angle of the fistula. It is of the greatest importance that the flap and the angles of the fistula should be both comprised in one noose of thread. The threads being passed, must be tied, and allowed to project externally. They separate in about fourteen days, but sometimes later. The urethral thread, which is attached to the summit of the flap, must be fixed in such a manner as to bear nothing during the movements of the patient. M. Jobert attaches it by diachylon plaster to one of the thighs.

A catheter must now be introduced into the bladder. If, in so doing, it come in contact with the flap, it must be turned on one side. A second catheter must be added to the first, to carry off the urine from the wounds which have been made. The catheter must be always open, and supported by cotton threads, attached to a bandage passing round the body. Constant repose in the horizontal position must be enjoined, in order that the bladder may not be irritated with the catheter; and that it may be retained as long as possible. The pedicle of the flap must remain undivided for a considerable period. M. Jobert divides it, between the thirtieth and fortieth day; but, in determining on the proper time, due regard must always be paid to the constitution and degree of vitality of the individual. The necessity of this delay is explained by the density of the tissues about the fistula.

As soon as the operation is terminated, some blood escapes into the bladder, and passes through the catheter: the same takes place in the vagina. The raw surface of the flap becomes covered with a layer of lymph; the urine is turbid, because of the pus which is secreted by its summit. This state of the urine varies as to its duration; enduring sometimes twenty-five days. More or less blood flows on the section of the pedicle, and one portion of it retracts towards the vagina, the other towards its root. It is liable to become inflamed from the contact of urine with its exposed surface; it swells, becomes red, and extends beyond the entrance of the vagina. Suppuration takes place, but, as this ceases, the inflamed portion retires gradually towards the vesico-vaginal septum. The flap entirely loses its sensibility: but the skin retains its structure, and hair grows upon it as before.

The experience which M. Jobert has hitherto had of the beneficial results of this operation, is founded on three cases; in two of which the operation succeeded; in the third, death took place in consequence of phlebitis; but, in the examination of the body, the union between the flap and the fistula was found to have established itself.

*Gazette Medicale.* Nos. 10, 13, 15. 1836.

---

*On the Cure of Erectile Tumours.* By Professor LALLEMAND, of Montpellier.

M. LALLEMAND was led, by observing the rapid cicatrization of an incision made in an erectile tumour during the partial removal of the lower jaw, and by reflecting on the complete obliteration by inflammation of portions of the corpus cavernosum and spongiosum, (to which erectile tumours are precisely analogous,) to attempt the cure of an erectile tumour, which from its situation could not be removed, by incisions and immediate union of the edges of the wounds. This tumour occupied the upper lip and extended into the nares; the first step consisted in removing, with two strokes of the scissors, a portion from the centre, of eight or ten lines in breadth: the wound bled freely, but the bleeding was immediately stopped by bringing the edges of the wound together, and fixing them with needles, around which thread was twisted as in the hair-lip operation. The wound healed favorably: the needles were removed on the fourth and fifth day, and the thread was detached on the twenty-fifth, leaving a solid cicatrix.

In about two months afterwards, M. Lallemand removed another portion in a similar way. As a proof of the success of the first cicatrix in partially obliterating the erectile tissue, it was observed that the blood merely oozed from the incision nearest to the first cicatrix, whilst it gushed freely from the opposite side; and the needles were passed with difficulty into the erectile tissue near the old cicatrix. The needles this time produced more abundant suppuration, but the wound healed, and its cicatrix, as well as those produced by the needles, looked like fibrous tissue. As the division of the nares, as well as the adjoining mucous membrane, remained tumefied, and as the needles produced a similar obliteration of the erectile tissue as the incision, M. Lallemand introduced them alone; and they produced the change he expected.

In a third case where incision was also employed, the effect of the needles in transforming the erectile into fibrous tissue was still more apparent, so that he employed in the next case needles alone.

An erectile tumour of three inches in length, two in breadth, and three lines in thickness, situated over the left scapula of a child of three months old, had been subjected to compression without advantage, and had doubled its size since birth. As the child was irritable, and very delicate, and the tumour was large, M. Lallemand was afraid to treat it by incision: he therefore introduced into the lower part of the tumour twelve fine pins, and covered the space which separated them with numerous circumvolutions of waxed thread. The child cried but little. Three days afterwards he made a similar application to the upper part, and attacked successively the whole circumference, leaving the pins for about seven or eight days, or even more, until they had produced sufficient inflammation. This occupied about forty days, and he was about to attack the centre, when he found it violet, tumid, and very hot; the general health was disturbed, and he suspended all treatment. To his surprise, the central part suppurated and collapsed, and in a fortnight was completely changed into a flat cicatrix. As some points of the circumference had escaped inflammation, it was necessary to repeat the introduction of the pins. After two months and a half of treatment, during which time 120 pins were introduced, the whole was converted into a pale fibrous tissue: not a teaspoonful of blood was lost, and the health of a delicate child was only slightly deranged for a few days.

Compression cures in a similar way, by producing inflammation: when compression is impossible or useless, this plan should be tried: it should also supersede the removal of the tumour, even when such an operation might be performed without danger or deformity. The stationary condition of such tumours does not warrant their not being operated on. The kind of operation must depend on the seat and extent of the disease. Where, as in the first case, the tumour arose from the alveolar border of the lower jaw, nothing better could be done than a removal of a portion of the bone, leaving undivided its lower border to prevent deformity, &c. Where the disease is very extensive, and occupies prominent and moveable parts like the lips, as it did in the second case, excisions and needles should be employed; but, where the erectile tissue is not free and moveable, the cure is best performed by exciting acute inflammation in it. Pins of a medium size are better than needles, as they can be cut with scissors, or their ends covered with forceps: the waxed thread is useless. Nitrate of silver, and probably nitrate of mercury frequently applied, keeps up the inflammation, which is the essential agent.

[Although our countryman, Dr. Marshall Hall, is fully entitled to the credit of priority in treating erectile tumours according to the principle advocated by M. Lallemand, (See Medical Gazette, Vol. VIII. 679,) it does not appear that the French Surgeon was acquainted with the practice recommended by Dr. Hall. At any rate the clear way in which he explains the steps by which he was led to substitute the insertion of needles for extirpation, renders his paper both valuable and instructive. Considerable credit is due to Mr. Abernethy for having recommended the simpler treatment by wet compresses and pressure, at the time when complete extirpation with the knife was practised generally. It is curious that he considered the increase of these tumours depended on inflammation, and that they were to be cured by subduing it.]

*Archives Générales de Medecine, Mai,* 1835.

*On certain Modifications in the Treatment of Hernia.* By M. Gerdy.

Professor Gerdy recommends the following plan of applying the taxis when the hernial tumour is so large that it cannot be grasped and pressed in its whole circumference; for in such cases the partial pressure does not become concentrated at the hernial opening, but pushes the whole mass in front of the ring.

He seizes between the extremities of the fingers of each hand that portion of the tumour nearest to the ring, at about an inch from the orifice, and, pressing the fingers together, he isolates this small portion from the rest of the tumour; he compresses it in its whole circumference, and by lateral movements he endeavours to return it. It generally requires only slight efforts to succeed, and the tumour is a little diminished; he then leaves the fingers of one hand applied to the ring, to prevent the reduced intestines from again protruding, and with the other hand seizes another portion in the same manner, and, abandoning the opening, he performs the same manipulation, but with greater facility; he continues in this manner until the hernia is so far reduced that he can grasp it with the hands, so as to compress it in its whole circumference, and thus reduce it as he would treat a smaller hernia. By this manipulation he has many times reduced hernias which have resisted the common methods, even when practised by experienced surgeons.

M. Gerdy also recommends the following modifications in the operation which he has practised as surgeon to the Hôpital Saint-Louis. After making the first incision through the integuments with a knife, he often completes the first part of the operation with straight probe-pointed scissors. The advantages consist in his operating more quickly, as he uses no director; in being able to distinguish better the parts raised on one of the blades of the scissors; in being more certain of cutting what he has seized, whilst, with a bistoury, the movement of the patient or assistant, or his own want of address, may cause the incision to be deeper than was intended. Scissors cut as well and clean as a knife, and, as it is only required to divide cellular or thin fibrous layers, there is no fear of that contusion which scissors are said to cause, the importance of which has been exaggerated.

M. Gerdy was induced by the following operation to invent a new form of knife for dividing the stricture. In operating for strangulated hernia, he found, after opening the sac, that the stricture was so narrow as to prevent his passing a director with the bistoury. He did not dare to pass a probe-pointed bistoury, flat, for fear of wounding the intestine. He then bent at a right angle, to an extent of two or three lines, the end of a silver director, and introduced its point. When he had passed the limits of the stricture, he depressed the body of the instrument perpendicularly to the trunk of the patient, so that the bent end of the sound was crooked round the posterior part of the ring, which he seized as by a hook. Then, whilst drawing the director gently towards him, he introduced into its groove a straight and acute bistoury, without fear of wounding any thing, as its point was lodged in the angle of the bent director, and the division of the stricture was easily effected. In consequence of this operation, M. Gerdy constructed a straight bistoury, about two lines wide in its whole length, terminated by a small cylindrical tongue, rather flattened, about one line in length, and united at a right angle to the end of the blade. The point of union was carefully rounded. Nothing can be more simple than the way in which it is used. The end of the tongue of the bistoury is introduced, guided by the finger, between the stricture and the strangulated mass: it passes as easily as a probe; the handle is then depressed, and the curved end of the bistoury passes up behind the ring. If, on depressing the handle, the instrument is instantly drawn towards the operator, it is impossible to wound the epigastric artery, even if it is in the way. To make more sure that the artery is not between the hook and the ring, the finger, whilst the operator draws the instrument to him, may be placed in the ring to ascertain if the pulsations are to be felt. The bistoury is then to be elevated, so as to divide by pressure the ring and corresponding portion of the sac.

*Archives génerales de Médecine. Avril,* 1836.

*On the Employment of Muriate of Barytes in the Treatment of White Swellings.* By M. LISFRANC.

The "Gazette Médicale" reports a clinical lecture of M. Lisfranc's, in which he relates the results of his experiments with this medicine, which has been long known, but has been recently brought into notice by M. Pirondi, of Marseilles.

Six grains of muriate of barytes are dissolved in four ounces of distilled water, of which one spoonful is taken every hour, except one hour before and two hours after each meal. In order to tolerate the medicine, the patient must abstain from wine and meat, taking only water and vegetable food. The bottle should not be exposed to the sun, or the salt will be precipitated, and the last spoonfuls contain a greater quantity: to avoid this, it should always be shaken. Sometimes the medicine produces slight pain in the stomach or a feeling of weight; but, if other symptoms do not follow, the stomach gradually becomes accustomed to the remedy, and the pain ceases. If, on the other hand, nausea, vomiting, or even some slight symptoms of poisoning come on, the medicine should be suspended, and cautiously resumed. The climate has some influence; for, although at Marseilles two drachms have been given, M. Lisfranc has never been able to increase the dose in Paris beyond forty-eight grains, and often he has been unable to reach that. The unpleasant symptoms have been removed by whites of eggs. Numerous patients have been submitted to this treatment, and the following are the conclusions which M. Lisfranc has arrived at.

1. Generally the white swelling has been much amended, and sometimes cured. 2. The benefit has been greatest amongst the scrofulous. 3. In some very few cases the muriate alone has cured. 4. After a certain time, the disease having become stationary, it was necessary to employ another method. At a later period, the renewed use of the muriate has produced excellent effects. 5. It may be employed both in the acute and chronic stage of white swellings. 6. Serious accidents have never resulted from its use; the slight symptoms before mentioned have always yielded readily. 7. A frequent effect is a diminution in the frequency of the pulse; this falling from sixty or eighty to forty or fifty, or even to twenty-five. 8. In some circumstances the medicine, continued at the dose of twelve grains during the month, has produced as much amendment as in other cases where the dose has been gradually augmented. 9. Where the patients have been slightly inconvenienced with the medicine, it has been most useful. 10. Compression and local abstractions of blood have been often combined with this treatment, and with extreme advantage.

M. Lisfranc considers muriate of barytes, given according to M. Pirondi's method, as a truly valuable acquisition to surgery, ("une vraie conquête chirurgicale.") *Gazette Médicale de Paris.* No. 14. 2 *Avril*, 1836.

---

*On the Cure of Intestinal Fistulæ by the Actual Cautery.* By Dr. FINGERHUTH.

THE success attending the employment of the hot iron in the cure of artificial anus, already recommended by Dieffenbach, is confirmed by two cases related by Dr. Fingerhuth.

In both, abdominal inflammation, caused by violent blows, had been followed by external abscess, to which succeeded discharge of fæcal matters. Various cauteries were employed to destroy the membranes lining the fistulæ, and to convert them into granulating surfaces, but without producing their complete obliteration. The fistulous openings, although somewhat diminished by imperfect granulations, showed no tendency to become closed. Cauterization was then adopted by means of an iron, corresponding in diameter to that of the fistulæ, and the temperature of which was scarcely elevated to that of red heat. Luxuriant granulations soon covered the cauterized parts, the fistulæ diminished, and the surfaces being again destroyed by a heated iron corresponding in size to the apertures which remained, they were eventually cured.

*Wochenschrift für die gesammte Heilkunde,* No. 6. 1836.

*Case of Local Hereditary Relaxation of the Skin.* By Dr. Graf, of Königsberg.

In his forty-seventh year, a strong man, the only exception to whose perfect health was a slight hæmorrhoidal complaint, first noticed a relaxation of the skin of the left side of his neck, and of the lower eyelids and the parts beneath them. The malady increased to such an extent, that in a few months the skin in these parts hung in the form of sacks; the skin being thin and covered with transverse and longitudinal furrows. The patient (a Russian) said that the malady was hereditary in his family. His grandfather, a year after his return from an imprisonment in Turkey, in which he had been exposed to great bodily and mental sufferings, began to be affected with the same disease, situated in the lower eyelids; and the deformity was so great, that he was obliged to confine himself entirely to the house. In his case the affection commenced in his forty-seventh year. He died at an advanced age, leaving two sons and a daughter. In her forty-fifth year, the daughter became the subject of the same malady, and, as she was unwilling to submit to an operation for its removal, she entered a nunnery, where she died very old. The elder brother was similarly affected in his forty-third year, the deformity being seated in the lower eyelids and left side of the neck. The parts were removed, and the disease did not return. The younger brother continued entirely free from the disease, and died in his seventy-first year. He left one son, the subject of the case which is here related.

The relaxed skin situated in the lower eyelids was operated on, in the same manner as a case of entropium. The wound healed by the first intention, and the deformity was removed. As the deformity of the neck could be conveniently concealed by the patient's dress, it was not removed by the knife. The patient was simply advised to apply to the part strong astringent remedies; and, in order to increase the tone of the whole skin, to use mildly astringent baths, as cool as possible. *Wochenschrift für die gesammte Heilkunde,* No. 15. 1836.

